# Post-mortem Characterisation of a Case With an *ACTG1* Variant, Agenesis of the Corpus Callosum and Neuronal Heterotopia

**DOI:** 10.3389/fphys.2019.00623

**Published:** 2019-05-24

**Authors:** Regina Vontell, Veena G. Supramaniam, Alice Davidson, Claire Thornton, Andreas Marnerides, Muriel Holder-Espinasse, Suzanne Lillis, Shu Yau, Mattias Jansson, Henrik E. Hagberg, Mary A. Rutherford

**Affiliations:** ^1^Centre for the Developing Brain, Division of Imaging Sciences and Biomedical Engineering, King’s College London, St Thomas’ Hospital, London, United Kingdom; ^2^Department of Neurology, University of Miami Miller School of Medicine, Miami, FL, United States; ^3^Department of Comparative Biomedical Sciences, Royal Veterinary College, London, United Kingdom; ^4^Department of Cellular Pathology, Guy’s and St Thomas’ NHS Foundation Trust, St Thomas’ Hospital, London, United Kingdom; ^5^Department of Clinical Genetics, Guy’s and St Thomas’ NHS Foundation Trust, Guy’s Hospital, London, United Kingdom; ^6^Perinatal Center, Department of Physiology and Neuroscience – Department of Clinical Sciences, Sahlgrenska Academy, University of Gothenburg, Gothenburg, Sweden

**Keywords:** heterotopia, radial glia, corpus callosum, growth cone, synaptic proteins

## Abstract

Cytoplasmic Actin Gamma 1 (*ACTG1*) gene variant are autosomal dominant and can cause CNS anomalies (Baraitser Winter Malformation Syndrome; BWMS). *ACTG1* anomalies in offspring include agenesis of the corpus callosum (ACC) and neuronal heterotopia which are ectopic nodules of nerve cells that failed to migrate appropriately. Subcortical and periventricular neuronal heterotopia have been described previously in association with ACC. In this case report, we investigated a neonatal brain with an *ACTG1* gene variant and a phenotype of ACC, and neuronal heterotopia (ACC-H) which was diagnosed on antenatal MR imaging and was consistent with band heterotopia seen on post-mortem brain images. Histologically clusters of neurons were seen in both the subcortical and periventricular white matter (PVWM) brain region that coincided with impaired abnormalities in glial formation. Immunohistochemistry was performed on paraffin-embedded brain tissue blocks from this case with *ACTG1* variant and an age-matched control. Using tissue sections from the frontal lobe, we examined the distribution of neuronal cells (HuC/HuD, calretinin, and parvalbumin), growth cone (drebrin), and synaptic proteins (synaptophysin and SNAP-25). Additionally, we investigated how the *ACTG1* variant altered astroglia (nestin, GFAP, vimentin); oligodendroglia (OLIG2) and microglia (Iba-1) in the corpus callosum, cortex, caudal ganglionic eminence, and PVWM. As predicted in the *ACTG1* variant case, we found a lack of midline radial glia and glutamatergic fibers. We also found disturbances in the cortical region, in glial cells and a lack of extracellular matrix components in the *ACTG1* variant. The caudal ganglionic eminence and the PVWM regions in the *ACTG1* variant lacked several cellular components that were identified in a control case. Within the neuronal heterotopia, we found evidence of glutamatergic and GABAergic neurons with apparent synaptic connections. The data presented from this case study with BWMS with variants in the *ACTG1* gene provides insight as to the composition of neuronal heterotopia, and how disturbances of important migratory signals may dramatically affect ongoing brain development.

## Introduction

Baraitser Winter malformation syndrome (BWMS) is associated with variants in the Cytoplasmic Actin Gamma 1 genes *ACTB* or *ACTG1*, which encodes β- and γ-actins (Verloes et al., 2014). Besides cerebrofrontofacial dysmorphisms, common CNS anomalies are pachygyria, subcortical band heterotopia and agenesis of the corpus callosum (ACC) (Verloes et al., 2014). The corpus callosum consists of over 200 million glutamatergic axonal fibers that connect the two cerebral hemispheres. Its formation requires intricate orchestration of numerous processes involving neuronal migration, synapse formation, and axonal guidance. The corpus callosal structure is formed by 20 weeks gestation and expands during antenatal development as its fibers are progressively premyelinated (Paul, 2011). ACC represents one of the most common antenatally diagnosed malformations of the brain and may be isolated or associated with other cerebral or non-cerebral abnormalities. Neuronal heterotopia has been reported in association with ACC, which may be clinically detected with MR imaging or only diagnosed histologically at postmortem. It is possible that neuronal migration disturbances are a common and unrecognized accompaniment of apparently isolated ACC. Neuronal migration is a well-orchestrated event. Neuroepithelial cells produce early radial progenitor cells in the ventricular zone and provide important cues for migrating neurons (Kadhim et al., 1993). The failure of neuronal migration due to genetic variants that interfere with glial structure and function are associated with the presence of neuronal heterotopias (Marcorelles et al., 2010; Kato, 2015; Ibanez and Andressoo, 2016). The presence of even small focal heterotopias in a cortical region can affect distant brain regions and give rise to behavioral abnormalities (Edwards et al., 2014). Neurodevelopmental impairments in isolated ACC are variable but are more severe in the presence of additional anomalies, and the occurrence of overt neuronal heterotopia is associated with poorer outcomes (Edwards et al., 2014; Ishii et al., 2015).

In the human embryo, callosal axons are first identified at 74 days post-conception, with adult morphology achieved around 115 post-conception days (Achiron and Achiron, 2001). In the frontal brain, the forceps minor fiber bundle, also known as the anterior forceps, connects the lateral and medial surfaces of the frontal lobes and crosses the midline via the genu of the corpus callosum. During development, the leading edge of the axonal fibers transverse into the genu using chemoattractant cues expressed by radial glia. The combination of neurons and glia are referred to as the indusium griseum glia (Sturrock, 1978). The indusium griseum glia forms the wide-spanning fibrous region of corpus callosum creating the callosal glial sling (Shu and Richards, 2001; Paul, 2011). In ACC, there may be a spectrum of developmental abnormalities from the entire absence of the corpus callosum to a thinning of callosal fibers that can be seen in the forceps minor.

In the developing brain, the cells of the subependymal germinative zone, a remnant of the subventricular zone, maintain high expression of antigen Ki-67 or MIB-1 for cellular proliferation (Moss et al., 2016) to support both radial glia and neuronal production. In later stages, 15–24 weeks gestation (Kostoviæ et al., 2015) of development, radial glia and phenotypically related astroglia are highly positive for vimentin or glial fibrillary acid protein (GFAP) (Bramanti et al., 2010; Moss et al., 2016). These radial glia will continue from 17 weeks gestation to guide the neuronal migration and the commissural axons of the corpus callosum (Edwards et al., 2014) by extension of the radial processes.

Neurons migrate to the cortex in a succession of waves using radial glia but follow two basic migration paths, either radial or tangential (Volpe, 2008). Radial neuronal migration is used by glutamatergic neurons (Lagercrantz, 2010) which, from 17–30 weeks gestation, will subsequently create axons that express a synaptosome-associated protein of 25 kDa (SNAP-25). SNAP-25 protein plays an important role in synaptic development and also maintains life-long callosal connections (Condliffe and Matteoli, 2011; Antonucci et al., 2013; Yang et al., 2017; Pozzi et al., 2018). The tangentially migrating neurons originate from the lateral, medial and caudal ganglionic eminence and translocate tangentially across the radial glial fibers also in a succession of migration waves (Erzurumlu et al., 2006; Sultan et al., 2014) to reach the cortex or into the central gray matter structures (Brazel et al., 2003; Wonders and Anderson, 2006). Neurons originating in the ganglionic eminence are mainly gamma-aminobutyric acid (GABA) ergic (Marin and Rubenstein, 2001; Kolasinski et al., 2013). GABAergic interneurons follow guidance cues from the extracellular matrix (ECM) and express distinct proteins that are specific to the region of origin. For instance, medial ganglionic eminence neurons are positive for parvalbumin, and somatostatin (Clowry, 2014; Sultan et al., 2014) whilst caudal ganglionic eminence (CGE) neurons express calretinin and neuropeptide Y (Wonders and Anderson, 2006).

Migrating neurons seek out environmental cues using a leading edge or growth cone structure (Leviton and Gressens, 2007; Volpe, 2008). The growth cone protrudes from the neuronal cell body as it leaves its site of origin (e.g., the ganglionic eminence) and elongates as it travels through the periventricular white matter (PVWM), using the radial glia as guideposts. The extended growth cone facilitates the neuron’s ability to migrate through the hyaluronic acid-rich ECM environment to reach its final destination (e.g., cortex) (Bruckner et al., 1993; De Luca and Papa, 2016). During migration or on contact with other neurons, the growth cone tip maintains its splay formation by using microtubule bundles and actin filaments (F-actin) and recruiting of another set of ligands, end binding 3 (EB3) and drebrin proteins (Geraldo et al., 2008; Conde and Caceres, 2009; Geraldo and Gordon-Weeks, 2009; Sonego et al., 2015). The effect of an *ACTG1* variant on growth cone structure and function is not well described.

This case study uses immunohistochemical analysis to investigate the corpus callosum (forceps minor and the genu) and the frontal cortex in a child subsequently diagnosed with an *ACTG1* variant and with clinical signs consistent with BWMS. To determine the pathophysiology associated with the *ACTG1* variant, we analyzed the underlying perturbation of migratory cues in the CGE by visualizing proliferation, neuronal and growth cone proteins. Additionally, we sought to find if the CGE of this case of with *ACTG*1 variant differed from the control in protein expression of radial glia and other types of glial cell (e.g., oligodendroglia and microglia). Finally, we completed a molecular dissection of the neuronal heterotopia seen within the PVWM, both on *in vivo* imaging and at histology, here, we sought to detect growth cone proteins, synaptic proteins and define neuronal composition (i.e., glutamatergic and GABAergic).

## Methods

Both written and informed parental consent was obtained from the participants of this study, acquired for post-mortem examination and post-mortem research according to National Health Service United Kingdom and Human Tissue Authority guidelines. Research study ethics was obtained from the National Research Ethics Service (West London), United Kingdom [ethics number, 07/H0707/139; Post-mortem Magnetic Resonance Imaging (MRI) Study of the Developing Brain].

### MRI

Magnetic Resonance Imaging of the fetal brains were acquired on a 1.5 Tesla Philips Ingenia scanner using single shot T2 weighted sequences acquired in three orthogonal planes using our standard clinical protocols. The case with the *ACTG1* variant was refrigerated (2–4°C) before post-mortem examination which was performed within 2 days of death. The whole post-mortem brain was fixed in 4% formalin for 5–6 weeks. Toward the end of the fixation period, MRI was performed on the fixed whole *ACTG1* brain at 3 Tesla (Philips), 21 days post-death.

### Tissue Preparation

This study assessed histological findings in the post-mortem brain of our *ACTG1* variant case where the pregnancy was terminated at 35.71 weeks. Findings were compared with an age-matched brain from a preterm infant who died (oligohydramnios and immature lungs) at 32.71 weeks gestational (GA); (age-matched control). The control infant brain showed no significant pathology on gross and microscopic examination and was, therefore, felt appropriate to be used as a non-neuropathological age-matched control (control case).

For both cases, the whole brain was sliced by a pathologist (A.M.), and the tissue blocks were processed on a Leica Tissue Processor (Leica Biosystems, Newcastle, United Kingdom). The paraffin-embedded tissue blocks were sectioned at 6 μm using a Leica RM2245 microtome (Leica Microsystems Ltd., Newcastle, United Kingdom). Paraffin-embedded tissue sections taken from the caudal frontal lobes (at the level of the posterior Ammon’s horn) were used for immunohistochemistry and histochemistry.

### Immunohistochemistry

Standard immunohistochemistry procedures for the brain sections have been described previously (Vontell et al., 2013, 2015) with the addition of methyl green counterstain being substituted for hematoxylin. Primary antibodies, catalog numbers, species and concentrates along with the secondary antibodies are listed in Supplementary Data.

### Histochemistry

The standard tissue paraffin block was sectioned at 6 μm and the slides were allowed to dry and then heated at 60°C for 30 min. Prior to staining, sections were deparaffinized in three changes of xylene and rehydrated through graded concentrations of ethanol. The histological stain, hematoxylin, and eosin (H&E), used to evaluate the general morphology of the tissue and orientation of the brain regions were described in Gill et al. (1974). The procedures for visualizing the extracellular matrix, the perineuronal nets, and hyaluronic acid content have been previously described (Bruckner et al., 1993) with the addition of Curtis Nuclear Stain (Leach, 1946).

### Microscopic Analyses

Unbiased images were obtained using the CM1 and CM2 modules for virtual tissue scan (MicroBrightfield Inc., Colchester, VT, United States) using stereology software (Stereo Investigator version 8.27; MicroBrightfield Inc.). The average area of each contour was encompassed by a 1.0–1.5-mm^2^ region, determined on the microscope using a 5× objective, to provide an average of 40 high-power field images per scan collected, using a 40× objective (0.0625 mm^2^) or a 63× objective (0.0594 mm^2^).

### Whole Exome Sequencing and Analysis

Whole exome sequencing (WES) capture was performed using Agilent SureSelect XT Clinical Research Exome (CRE; SureSelectXT Human All Exon V5; Santa Clara, CA, United States) baited with clinically relevant genes. The enriched exome libraries were sequenced using paired-end, 125 cycle chemistry on an Illumina NextSeq 550 (Cambridge, United Kingdom). An integrated laboratory and bioinformatics platform was used in which Agilent CRE libraries are sequenced with automatic data transfer to DNAnexus for alignment and variant calling using BWA and GATK. The quality threshold was set at 95% at 20X coverage. The generated VCF files were uploaded into QIAGEN Ingenuity Variant Analysis (IVA; Manchester, United Kingdom) for assessment. The use of in-house designed “virtual panels” or 100K Genomes “PanelApp” gene panels restricts the analysis to only clinically relevant genes. Assessment of pathogenicity was performed using Performed using QIAGEN IVA (CADD, CentoMD, EVS, Allele Frequency Community, JASPAR, Ingenuity Knowledge Base, Vista Enhancer, OMIM, gnomAD, Clinical Trials, BSIFT, TCGA, PolyPhen-2, 1000 Genome Frequency, Clinvar, DGV, COSMIC, ExAC, HGMD, PhyloP, DbSNP, TargetScan, SIFT4G), and Alamut for splice site analysis (Rouen, France; SpliceSiteFinder-like, MaxEntScan, NNSplice, GeneSplicer). Variant classifications were performed according to ACMG guidelines (Richards et al., 2015). The case was then presented at a mixed disciplinary team meeting where the genetic findings were discussed in light of the clinical findings and the phenotype of the patient. A diagnostic report was issued, alongside a list of all genes analyzed within the panel, detailing the coverage at 20X.

## MRI Findings

**FIGURE 1:**
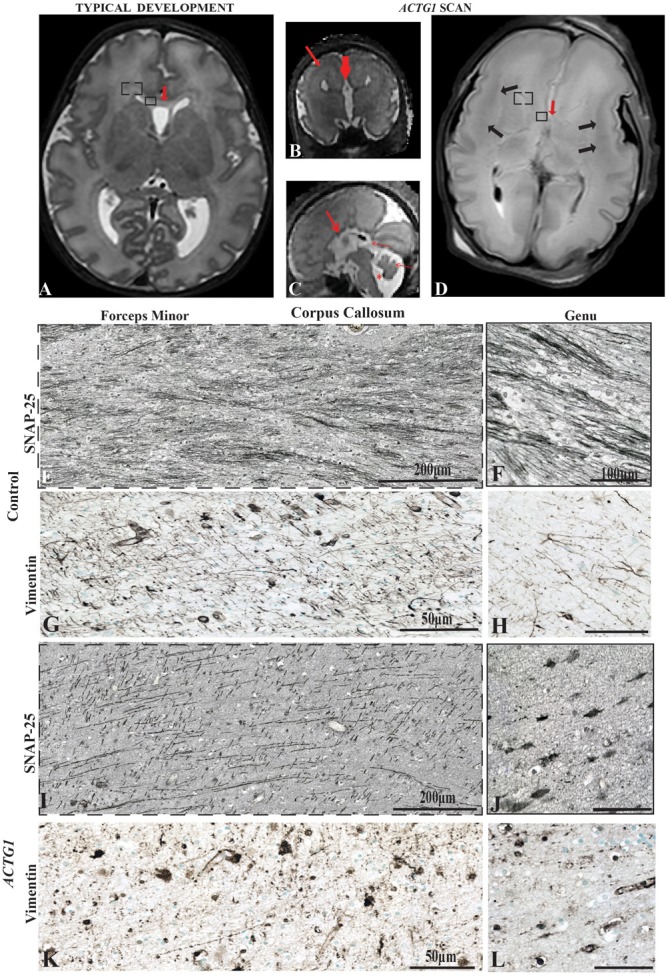
Magnetic Resonance Imaging axial plane using T2 weighted images. In the transverse orientation, the scans show normal anatomical characteristics of a fetal brain at 35 GA wks **(A)**. Coronal T2 weighted image of the case with a variant in the *ACTG1* gene and agenesis of the corpus callosum with neuronal heterotopia (ACC-H) at 35.30 weeks showing the absence of the corpus callosum (thick red arrow) and prominent low signal intensity band in the subcortical white matter (thin red arrow; **B**). The sagittal T2 weighted image of the *ACTG1* variant, shows the absence of the corpus callosum (thick red arrow) and shortened cerebellar vermis, rotated away from the brainstem (thin red arrow) giving an enlarged fourth ventricle (asterisk; **C**). The genu of the corpus callosum is seen in panel (**A**; red arrow) but is absent in the case with agenesis of the corpus callosum (*ACTG1* variant, **D**, red arrow). On post-mortem imaging, the brain in the fetal case with *ACTG1* variant **(D)** showed an absence of the corpus callosum and decreased cortical folding frontally. There is extensive bilateral abnormal low signal intensity within the subcortical white matter (black arrows) on the T2 weighted MRI. Immunostaining of SNAP-25 **(E,F,I,J)** and vimentin **(G,H,K,L)** from the forceps minor **(E,G,I,K)** and the genu **(F,H,J,L)** of the corpus callosum of the frontal lobe (represented as a box-in region in images **A,D**). Image E shows normal axonal fibers in the control, whereas, in the photomicrograph I the axons are not as numerous and have unusual tangential fibers seen in *ACTG1* variant. Images **(F,J)**, show SNAP-25 positive fibers in the genu of the corpus callosum from the control case **(F)** which are severely reduced in ACC-H **(J)**. The callosal fibers rely on the midline glial structures to serve as guidance mechanisms. Image **(G)** shows normal vimentin positive indusium griseum glia (IGG) that guide the callosal axons of forceps minor. The horizontal IGGs are punctate in the *ACTG1* variant **(K)**. Callosal fibers cross the hemisphere by following tracts laid out by the glial wedge as seen in the control **(H)** which are absent in the *ACTG1* variant **(L)**. Scale bar in images **(H,J,L)** = 100 μm.

In this case study, we investigated a post-mortem brain 35.71 gestational age (GA; weeks). The mother (gravida 2 para 1) presented with a female fetus demonstrating both fetal growth restriction and signs consistent with ACC on an ultrasound conducted at 33 GA weeks. A fetal MRI conducted at 35.30 GA weeks confirmed the ultrasound diagnosis of ACC (Figure 1B) and associated colpocephaly, bilateral posterior dilatation of the lateral ventricles. The anterior commissure was thicker than usual, whereas the posterior commissure was only just visualized. In addition, there was incomplete operculisation of the Sylvian fissures. The MRI confirmed a small head circumference (<1st centile) and also detected a small cerebellar vermis (height < 1st centile), which was rotated away from the brainstem in the midline sagittal views, consistent with cerebellar hypoplasia (Figure 1C). The cerebellar hemispheres and brain stem appeared normal, but both the transcerebellar diameter and pontine brainstem diameter were < 1st centile. The frontal aspect of the brain appeared narrowed, and the frontal white matter demonstrated bilateral regions of abnormal low signal intensity on T2 weighted images (Figure 1B) which are seen in typical development (Figure 1A). The latter could not be more formally assessed as image quality was suboptimal. The parents decided to terminate the pregnancy at 35.71 GA.

The post-mortem MRI of the brain confirmed the antenatal findings of microcephaly, absent ACC and incomplete operculisation of the Sylvian fissure. In addition, there were extensive low signal intensity bands in the subcortical white matter on the T2 weighted images, consistent with band heterotopia (Figure 1D) and more marked than appreciated on the antenatal MRI.

**FIGURE 2:**
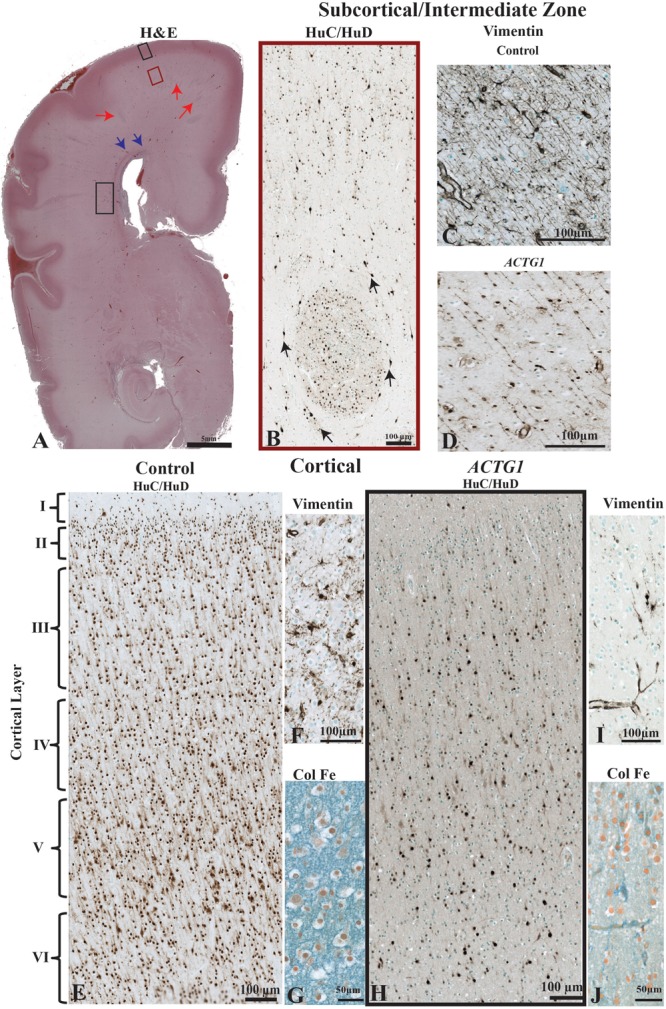
The morphological differences between the age match control and the case with *ACTG1* variant, neuronal heterotopia, and agenesis of the corpus callosum. In image **(A)**, a coronally oriented scan from Haematoxylin and Eosin (H&E) staining of the frontal lobe of the brain of the *ACTG1* variant shows neuronal heterotopia in the subcortical, intermediate zone white matter (red arrows) and in the periventricular white matter regions (black rectangle). The blue arrows in image **(A)**, are pointing to a region where there are severe disruptions of the callosal fiber tracts. In image **(B)**, the immunoreactivity of anti-HuC/HuD from the subcortical region (from the red-boxed region on image **A**) shows an example of a whirling heterotopia with neurons located in and around a nodular structure. The black arrows are pointing to neurons adjacent to the heterotopia. Image **(C)**, show the immunoreactivity from using anti-vimentin, which identifies radial glia and astrocytes in the control. In the *ACTG1* variant **(D)**, the vimentin-positive radial glia are fragmented and sporadically situated. The black boxed seen in image **(A)** in the rostral frontal cortical region from the *ACTG1* variant is exemplified in image **(H)** using anti-mouse HuC/HuD. This image shows the poorly distribution of cortical neurons compared to the control in image **(E)**. Anti-vimentin staining of the cortex **(F,I)** show normal radial glia fiber end (**F**; control) which are nearly absent (**I**; *ACTG1* variant). Additional differences can be seen in the extracellular matrix in the cortex using the Colloidal Iron Stain. Image **(G)** shows a vast perineuronal network with hyaluronic acid complexes, which is not as dense in the *ACTG1* variant **(J)**.

The microscopic examination of the brain confirmed that this infant had evidence of abnormal neuronal migration with subcortical and PVWM neuronal heterotopia (Figure 2A, 6C) and findings also confirmed ACC-H. The clinical post-mortem examination also detected a severely hypoplastic left kidney. The heterozygous *ACTG1 gene* variant [ACTG1 NM_001614.3 c.608C > T p.(Thr203Met)] was subsequently confirmed as a Pathogenic Class 4 (PS3) (Richards et al., 2015) based on the interpretation of the genetic and clinical findings and the phenotype of the patient.

## Results

These experiments were designed to assess the pathophysiological effect of the *ACTG1* variant by demonstrating differences between the gene variant and an age-matched control in the regions of the corpus callosum, frontal cortex, intermediate zone, CGE and in the PVWM. We also sought to examine the composition of the neuronal heterotopia (35.71 GA weeks) and the surrounding support structures (i.e., the germinal matrix and the radial glia).

### Abnormal Midline Radial Glia and SNAP-25 Expression Is Seen in ACTG1 Variant

During development of the corpus callosum, the leading edge of the commissural axonal fibers expresses the SNAP-25 protein. The axons transverse into the genu by chemoattractant cues in the vimentin-positive radial glia of the early corpus callosum referred to as the callosal glial sling between 17 and 30 weeks gestation (Shu and Richards, 2001).

In ACC, there is a spectrum of developmental abnormalities ranging from the entire absence of the corpus callosum to conditions where the callosal fibers may have started to grow, but when unable to cross between the hemispheres, they grow toward the back of the same hemisphere where they began termed Probst bundles (Hetts et al., 2006).

We investigated the axonal fibers and the glial sling in two respective regions of the corpus callosum, the forceps minor connecting lateral and medial surfaces of the frontal lobes (Figure 1E,G,I,K) and the genu (Figure 1F,H,J,L) in both the control (Figure 1E–H) and the case with an *ACTG1* variant, aged 35 weeks (Figure 1I–L).

In the control case, the forceps minor had numerous SNAP-25 positive fibers (Figure 1E) that stretched across the hemisphere and into the genu (Figure 1F). The radial glia in the forceps minor were visualized using anti-vimentin (Figure 1G). Remnants of vimentin indusium griseum glia were still present in the genu (Figure 1H) which is considered normal in the third trimester of fetal development (Marcorelles et al., 2010).

The anti-SNAP-25 axonal fibers in the *ACTG1* variant were thicker and more sparse with perpendicular pointing fibers (Figure 1I) in the forceps minor but showed a more coiled shape in the genu (Figure 1J). The vimentin immunoreactivity showed different morphology in the *ACTG1* variant from that of the control. In the forceps minor and the genu of the control there were fibrous elongated radial glia (Figure 1G,H), but in the *ACTG1* variant, there were cuboidal-shape astroglia (Figure 1K,L). These findings suggest that the indusium griseum glia of the corpus callosum failed to develop and lacked the SNAP-25 positive axonal fibers which are normally guided into the forceps minor.

### Heterotopia Are Seen in the Subcortical Regions of the ACTG1 Variant

The MRI showed extensive bilateral low signal dense bands consistent with neuronal heterotopia in the case with *ACTG1* variant within the frontal-parietal cortical regions: these were absent in the control cases. We examined the cellular composition of the subcortical region at the level of the posterior Ammon’s Horn (Figure 2A). Amongst the migrating neurons seen with the neuronal marker HuC/HuD, the *ACTG1* variant revealed whirls of densely positive neuronal heterotopia (Figure 2B). The heterotopia were absent in the control (not shown).

We then investigated the morphology of the radial glia in the intermediate zone using vimentin. In the control case, the linear radial glia were identified extending into the cortical regions (Figure 2C). The *ACTG1* variant had disjointed vimentin-positive extensions (Figure 2D) with nodular cell bodies.

Since the subcortical white matter had multiple heterotopia consistent with a band seen in the MRI, we investigated the morphology of the adjacent cortical region. We compared the neuronal and astroglial populations within the cortex seen in the control with those seen in the *ACTG1* variant. The control case demonstrated a six-layered isocortex with numerous neurons (Figure 2E) and astrocytes (Figure 2F) seen in each layer.

In the *ACTG1* variant, the cortical region was only sparsely positive for the neuronal marker HuC/HuD (Figure 2H). The vimentin-positive astroglia were only spread scantily throughout the cortex (Figure 2I) and was there was a deficit in cell numbers across all cortical layers.

We next assessed the ECM in the cortical region using the Colloidal Iron Stain. In the control the ECM was rich with hyaluronic acid components and was densely positive in the control (Figure 2G); this was absent in the *ACTG1* variant (Figure 2J) case.

The deficits of ECM in the cortex and the underlying white matter demonstrate that the neuronal environment may not be not conducive to structures that require hyaluronic acid components to facilitate their ability to navigate and therefore it may not be supportive of neuronal/glial migration and positioning within the cortex.

### Deficits of Neuronal and Astroglial Proteins Are Seen in the CGE in the Case With ACC-H

The cellular morphologies seen in the cortical, subcortical and intermediate structures in the *ACTG1* variant (Figure 2) showed many histological abnormalities that could have been due to disturbances in radial glia or the ECM composition.

However, it is also possible that these changes were due to primary abnormalities in the germinal zones; the subventricular zone and the ganglionic eminence, the latter being highly active during mid-late fetal gestation. We identified cortical abnormalities in the *ACTG1* variant which may have been due to the disturbances seen in the radial glial or the lack of proliferation signals stemming from the subventricular zone in the early stages of development.

Therefore, in the next series of experiments, the distribution of proliferation markers, neuronal cells, and growth cone proteins were investigated in the CGE as this region is still highly active around 35 GA weeks. MIB-1 (Ki-67) antibodies are commonly used to identify proliferation densities in sections prepared with paraffin processing (Mathews et al., 2017).

**FIGURE 3:**
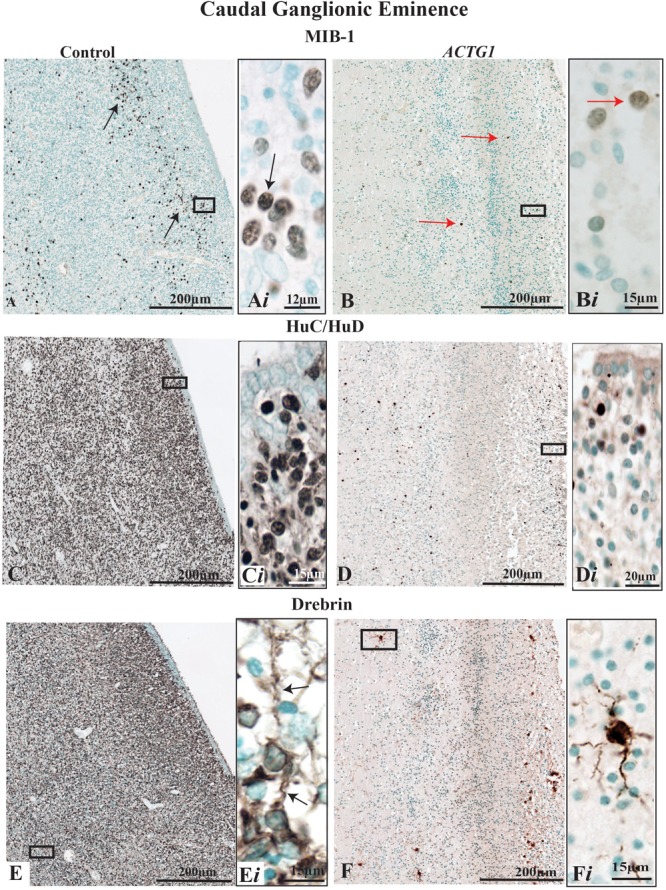
Immunoreactivity of cell proliferation (MIB-1) neurons (HuC/HuD) and growth cone structures (Drebrin) from the caudal ganglionic eminence (CGE) of a control case (32 GA wks; **A,C,E**) and the case with heterotopia and agenesis of the corpus callosum (*ACTG1* variant; 35 GA wks; **B,D,F**). In image **(A)**, the black arrows are pointing to positive cells for mouse anti-MIB-1 (ki-67) and demonstrate a reasonable distribution of proliferating cells **(A*i*)**. Whereas, in image **(B)**, the red arrows are pointing to the sparse population of proliferative cells in the *ACTG1* variant that are not as strongly stained **(B*i*)**. HuC/HuD immunoreactivity shows the neuronal population in the CGE is seen in the control brain **(C,C*i*)** and in *ACTG1* variant **(D,D*i*)**. Drebrin positive, growth structures are seen in images **(E,F)**. The control brain is densely packed with growth cones **(E)** and the inset shows elongating extensions arising from the cell (black arrows; **E*i***). The *ACTG1* variant has neurons that are positive for drebrin **(F)**, but the cell shown in the inset **(F*i*)** exemplifies that the staining is dense and fragmented rather than perinuclear with long extensions.

MIB-1-positive cells were less densely stained and not as numerous in the *ACTG1* variant (Figure 3B,Bi) compared with the control (Figure 3A,Ai). The lack of MIB-1 protein translated to a decrease in neuronal cells within the CGE shown by the reduced number of HuC/HuD positive cells in the *ACTG1* variant (Figure 3D,Di) compared with the control (Figure 3C,Ci).

Parallel microtubule bundles with MAP proteins (i.e., MAP1B or MAP2) are captured in the growing axons and dendrites via linking-proteins, such as Drebrin (Conde and Caceres, 2009). Drebrin is localized in two regions of the growth cone, in the T-zone and the filopodia. Drebrin protein is essential for forming links with EB3 to solidify synaptic connections and is required for neuritogenesis (Ivanov et al., 2009a,b). Although prominent drebrin leading edges were seen on the neurons in the CGE (Figure 3Ei) the number of drebrin positive cells was reduced in the *ACTG1* variant (Figure 3F,Fi) compared with the age-matched control (Figure 3E,Ei).

The ganglionic eminence relies on cells of the neuroepithelia to line the ventricles to support and protect the adjacent tissue in the lateral ventricles from edema. Radial glia and ependymal cells arise from the same lineage of neuroepithelial cells after the onset of neurogenesis. Radial glia guide migrating neurons while ependymal cells line the cavities of the CNS to help circulate the cerebrospinal fluid. We extended our investigation of the CGE to include the protein expression of nestin, vimentin, and GFAP as these are vital to the survival of the germinal matrix function.

**FIGURE 4:**
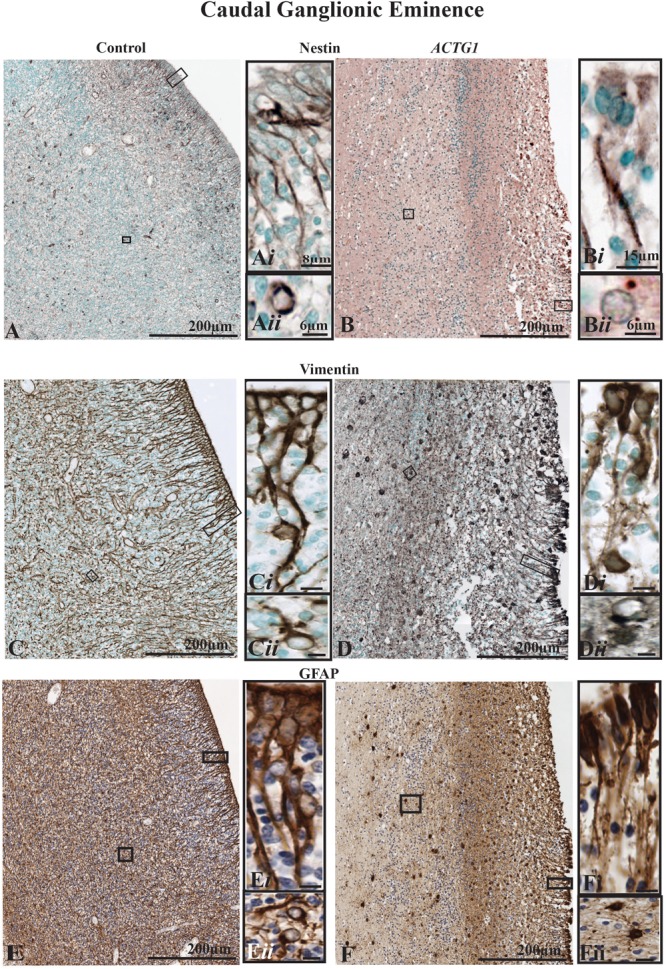
The radial glia proliferation and filament marker, nestin (images **A,B**), and the radial glia and astroglia markers [vimentin (images **C,D**) and GFAP (images **E,F**)] in the caudal ganglionic eminence (CGE). Immunoreactivity of nestin in the control brain **(A)** demonstrates a normal distribution along the ependymal cells that develop from tanycytes, types of transitional cells with radially extending processes that extend from the lining of the ventricle **(A*i*)** into the lateral ganglionic eminence. Image **(A*ii*)** shows a newly form astrocyte, that was probably migrating out of the CGE with perinuclear nestin expression. The ependymal lining is fragmented in the case with heterotopia and agenesis of the corpus callosum (ACC-H; images **B,D,F**) and the nestin-positive radial glial are sparse and lack the long extending processes **(B*i*)** additionally astroglia seen in the CGE do not have strong perinuclear staining **(B*ii*)**. The mouse anti-vimentin and the mouse anti-GFAP immunostaining show a similar pattern to the Nestin in the cases. In the control case **(C,E)** there densely packed radial glia and the fibers are strongly positive for vimentin **(C,C*i*)** and GFAP **(E,E*i*)**. Immature astrocytes are seen in the CGE **(C*ii*,E*ii*)** with stout processes. Whereas, in the *ACTG1* variant **(D,F)** the radial glia fibers are thin **(D*i*,F*i*)** and fragmented as they emerge from the ventricular lining. In the CGE of the *ACTG1* variant, there are dense vimentin and GFAP positive cuboidal gliotic astrocytes **(D*ii*,F*ii*)**. Scale bar in images **(C*i–*F*ii*)** = 8 μm.

The control demonstrated normal astroglial cell histology as along the ventricular wall, the cell bodies formed tight gap junctions, and in the CGE the astroglia were typical star-shaped (Figure 4A–Aii,C,E). With the *ACTG1* variant, nestin expression was remarkably decreased along the ventricle (Figure 4B–Bii) compared to the control. In the control (Figure 4C,D), the cell bodies of the glia had radial processes with long protruding ends (Figure 4Ci,Ei) which were identified with both vimentin and GFAP proteins. The vimentin and GFAP expression in the CGE were similar in morphology in the *ACTG1* variant (Figure 4D,F) and showed gaps between the cell bodies and the protruding end that normally forms the radial glia (Figure 4Di,Fi) were not as prominent.

The dense expression of vimentin and GFAP seen in the PVWM (adjacent to the CGE) in the *ACTG1* variant is indicative of astrogliosis (Figure 4Dii,Fii), this pathology was not seen in the control (Figure 4Cii,Eii). These finding suggests that in this case with *ACTG1* variant, deficits in the CGE may be related to a lack of migration cues and reduced expression of proliferation proteins.

### The Expression of Oligodendroglia and Microglia Proteins Are Decreased in the ACTG1 Variant

**FIGURE 5:**
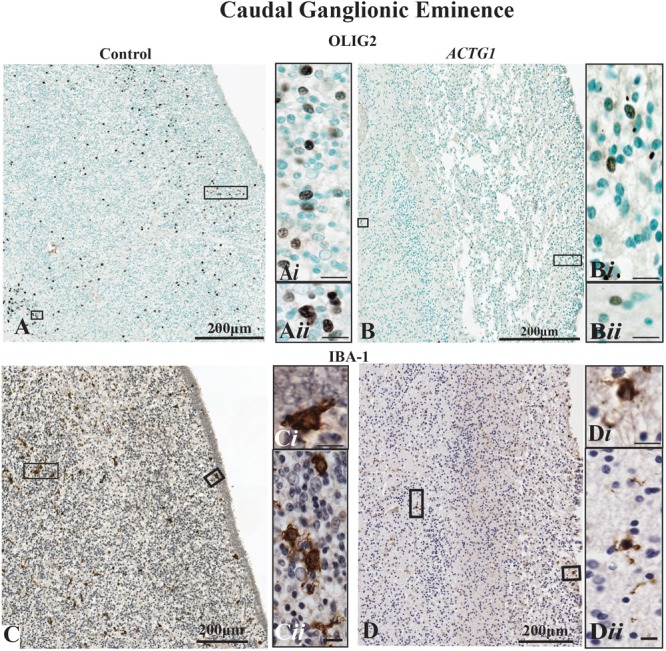
Oligodendroglia and microglia populations shown with markers for mouse anti-OLIG2 and rabbit anti-Iba-1. In the control case (image **A**) the OLIG2 positive cells are dispersed throughout the caudal ganglionic eminence (CGE), and the insets show densely OLIG2 positive nuclei **(A*i*,A*ii*)** found throughout the CGE. Image **(B)** demonstrates that the OLIG2 positive nuclei in the CGE lack in the heterotopia and agenesis of the corpus callosum (ACC-H) case (insets **B*i*,B*ii***). The immunoreactivity of rabbit anti-Iba-1 shows ameboidal microglia **(C)** are seen in the beneath the ependymal cells **(C*i*)** of the CGE and in regions adjacent to the white matter **(C*ii*)** of the control case. The *ACTG1* variant **(D)** show Iba-1 immuno-stained microglia beneath the ependymal cells **(D*i*)**, but in the CGE adjacent to the white matter the microglia in the *ACTG1* variant are much smaller **(D*ii*)**. Scale bar in inset images = 9 μm.

As we saw a change in the astroglia morphology in the *ACTG1* variant case, we sought to determine if other glial populations were affected. Hence, we stained for oligodendroglia using anti-OLIG2 and microglia with anti-Iba-1. In the CGE of the control case, there was a moderate to frequent expression of the OLIG2 protein (Figure 5A–Aii). Although oligodendrocytes are present in the *ACTG1* variant, OLIG2 immunolabeled cells labeled with mouse anti- OLIG2 immunoreactivity were scant (Figure 5B–Bii). Surprisingly, with edema in the *ACTG1* variant seen around the ventricular endothelial and white matter regions, there was only a low level of Iba-1 expression (Figure 5D–Dii). The control had ameboidal Iba1-positive microglia seen along the ventricular lining (Figure 5C,Ci) and in the CGE regions that were adjacent to the white matter (Figure 5C,Cii). The absence of oligodendroglia and microglia may indicate that there may be significant deficits in this brain with heterotopia which may interfere with the migrating neurons and the proliferation of supporting glia; how this attributes to the formation of the heterotopia remains unclear.

### White Matter Neuronal Heterotopia Are Positive for Synaptic Proteins

Since we identified a decrease of neurons in the CGE and clusters of heterotopias in the subcortical region of the *ACTG1* case, we next assessed the PVWM (black rectangle in Figure 2A).

**FIGURE 6:**
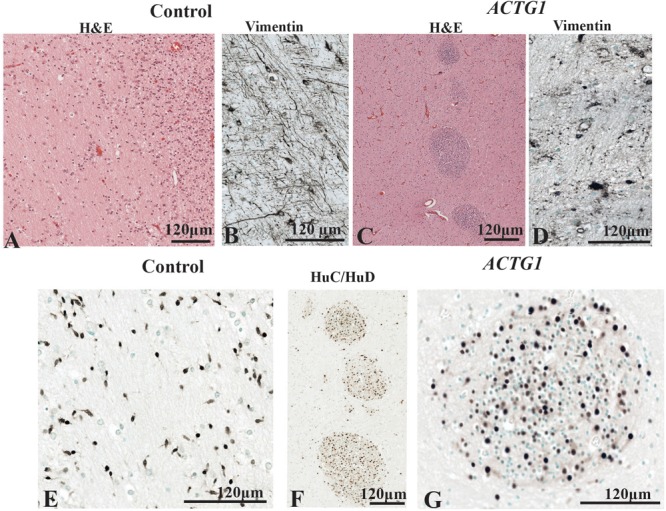
The periventricular white matter (PVWM) adjacent to the caudal ganglionic eminence (CGE; see Figure 2A). In the control, the hematoxylin stain shows the normal morphology of the PVWM **(A)** whereas in the case *ACTG1* variant and heterotopia and agenesis of the corpus callosum (ACC-H) cellular clusters are found instead white matter fibers **(C)**. Inside the control **(B)** case there are linear radial glial along with perpendicular astroglia seen with anti-vimentin, however, in the *ACTG1* variant, the astroglia processes are disjointed **(D)**. The HuC/HuD immunostaining in the control case demonstrates migrating neurons within the PVWM **(E)** in the control case. Images **(F,G)** show that the cellular clusters are neuronal heterotopia seen the *ACTG1* variant using mouse anti-HuC/HuD.

The PVWM region adjacent to the CGE is an important part of the paracentral stratified transitional field and migratory stream and was investigated using H&E, anti-Vimentin and anti-HuC/HuD antibodies and. In the control case (Figure 6A,B,E) there were regular patterns of white matter fibers (see Figure 6A). Amongst the fibers were vimentin-positive astroglia and radial glia (Figure 6B) that either followed the white matter tracks or lay perpendicular to them. The HuC/HuD immunostaining in the control demonstrated that the white matter fibers house migratory streams of neurons well into the third trimester of gestation (Figure 6E).

In the *ACTG1* variant (Figure 6C,D,F,G) the H&E shows the morphology of the PVWM fibers that were less uniform, and there were round clusters of cells (Figure 6C). Immunostaining with the mouse anti-HuC/HuD antibody identified the cells in the clusters to be neurons (Figure 6F,G) consistent with neuronal heterotopia. Dense vimentin-positive astroglia were seen in the PVWM regions of the *ACTG1* variant (Figure 6D) and around the heterotopia (Supplementary Data), however, the astroglial processes were fragmented.

### PVWM Neuronal Heterotopia Have Both GABAergic and Glutamatergic Neurons

We identified that the heterotopia contained neurons (i.e., HuC/HuD positive cells) therefore we sought to determine if the neurons were making synaptic connections, using the markers for growth cones (drebrin), synaptic processes (synaptophysin and SNAP-25) and interneuronal markers (calretinin and parvalbumin).

**FIGURE 7:**
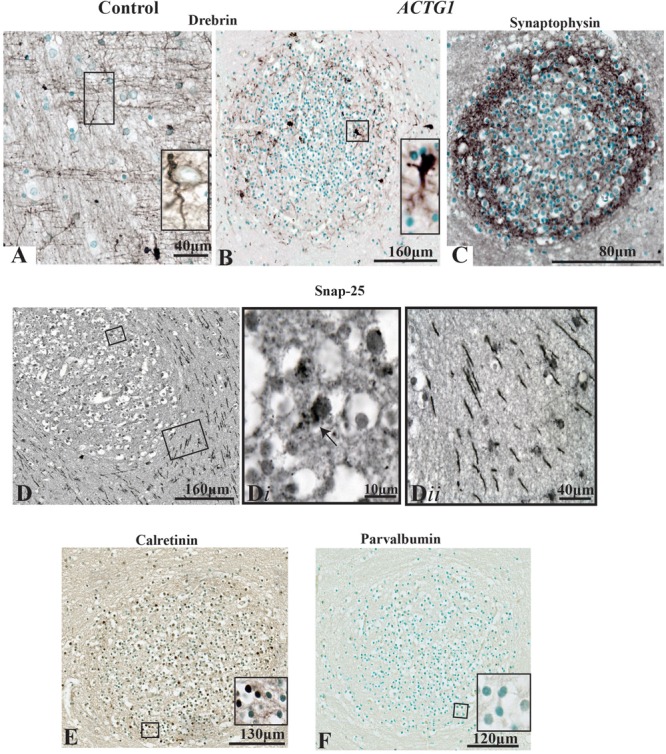
Photomicrographs of the periventricular white matter (PVWM) region adjacent to the caudal ganglionic eminence (CGE; outlined in Figure 2) demonstrating the growth cone proteins (anti-drebrin), synaptic connections (anti-synaptophysin and anti-SNAP-25) and interneuronal proteins (anti-calretinin and anti-parvalbumin). In the control case **(A)** anti-drebin expression is seen on growth cones as long bipolar extensions. In contrast, the case with *ACTG1* variant, heterotopia are identified with neurons expressing dense drebrin protein with blunt and short extensions **(B)**. The immunoreactivity of synaptophysin densely surrounds the outer regions of the neuronal heterotopia **(C)**. Detection of glutamatergic cells inside the neuronal heterotopia is seen with anti-SNAP-25 staining **(D,D*i*)**. Of interest, the PVWM region surrounding the neuronal heterotopia have SNAP-25 positive axons **(D*ii*)**. Additionally, rabbit anti-calretinin neurons seen in the neuronal heterotopia **(E)** however, the neuronal heterotopia were negative for rabbit anti-parvalbumin **(F)**.

In the PVWM of the control case, the neuronal growth cones had long leading drebrin-positive ends (Figure 7A). Although the neurons inside the heterotopia expressed drebrin, the fine extensions seen in the control are lacking, and the expression is more dense and centered around the nucleus (Figure 7B). Synaptophysin, a marker for synaptic vesicle transmission, was identified around the perimeter of the heterotopia (Figure 7C). However, toward the center, the staining was more sporadic. Dense perinuclear and axonal SNAP-25 processes were identified within the heterotopia which may be indicative of glutamatergic neurons (Figure 7D,Di). Of interest, linear SNAP-25 protein expression was also seen around the heterotopia which indicates that axons circumnavigate the heterotopia (Figure 7Dii). In addition, GFAP and Iba-1 immunoreactivity also circumvented the heterotopia (Supplementary Data).

Finally, we used the interneuron markers calretinin and parvalbumin to identify any GABAergic neurons within the heterotopia. In the PVWM heterotopia, we identified calretinin-positive neurons (Figure 7E), but these were negative for parvalbumin proteins [Figure 7F; although parvalbumin fibers and neurons were seen in the putamen (see Supplementary Data)]. The significance of calretinin positive neurons opposed to parvalbumin neurons may be indicative of the origin of neurons seen in the heterotopia (i.e., the CGE) (Wonders and Anderson, 2006). Nevertheless, the data presented in this section show that within the heterotopia of this *ACTG1* variant, the neurons may have made synaptic connections and may be comprised of both glutamatergic and GABAergic neurons.

## Discussion

In this case report, we have described deficits in the expression of essential proteins in a near-term fetus with a variant in the *ACTG1* gene and demonstrating microcephaly and ACC-H by comparing this case with an age-matched control.

We have shown that in this case of the *ACTG1* gene variant, there are disruptions in radial glia, which are comorbid with a loss of axonal fiber density in the corpus callosum. Additionally, we found disturbances in the subcortical and intermediate zone radial glia composition and a dramatic decrease in cortical neurons In the *ACTG1* variant, there is a marked lack of proliferative signals (MIB-1) and guidance structures (growth cones proteins and glia), which are normally found in the caudal ganglionic eminence. Moreover, these findings imply that neuronal heterotopia are more complex as we show that they are composed of both glutamatergic and GABAergic neurons. To the best of our knowledge, this is the first time that synaptic proteins, growth cone structures, and axonal morphology in and around neuronal heterotopia have been shown in the human neonatal brain.

The results presented in this study of an *ACTG1* variant, are compatible with other case studies that analyzed neuronal migration disturbances in type I lissencephaly (*LIS1*), doublecortin (*DCX*) and aristaless-related homeobox gene (*ARX*) variants (Ross et al., 2001; Paul et al., 2007; Marcorelles et al., 2010). *DCX* and *LIS1* genetic variants cause a very similar phenotype of subcortical heterotopia. Although *LIS1* variants have been associated with isolated lissencephaly, where the parietooccipital brain region is more affected, *DCX* variants often give rise to malformations of the frontal cortex. Furthermore, variants in *DCX* result in lissencephaly in males and subcortical laminar heterotopia in females (Watrin et al., 2015). Variants in the *ARX* gene are characterized by abnormalities such as microcephaly and lissencephaly, plus ACC (Marcorelles et al., 2010). The specific mechanisms that relate ACC and neuronal heterotopia are still unknown, although other investigations show that *LIS1* and *DCX* deletions result in radial glial depletions and microtubule malfuctioning (Watrin et al., 2015). Recently, other candidates such as glial cell line-derived neurotrophic factor (GDNF) and the GDNF family receptor alpha-1 have shown that variants, even when heterozygous, can upset the proliferation of radial glia and disturb migration (Ledda et al., 2007) and synaptogenesis (Ibanez and Andressoo, 2016).

In this post-mortem, near-term fetal study, we showed microcephaly, ACC with comorbid heterotopia seen subcortically and in the PVWM. Furthermore, there was a decrease in cortical volume and radial glia structures compared with the aged-matched control. These findings imply that other supporting glia may be perturbed which would have serious consequences to the developing brain.

The GA at birth of the age-matched control was similar to the *ACTG1* variant, but we cannot exclude the possibility that the control would have developed neuronal pathologies if it had lived longer, however, this is highly unlikely as these are early developmental changes and one would have expected to see them. Nevertheless, our data suggest that compared to the age-matched control the mechanism underlying overt pathophysiological changes seen in *ACTG1* variants are related to radial glia disturbances that affect GABAergic and glutamatergic neuronal migration.

### Neuronal Proliferation and Pathological Findings

Previous studies focusing on subcortical neuronal heterotopia have shown relationships between cortical lamination and the genetic abnormalities associated with the mechanisms of migration impairment in agyric/pachygyria syndromes (Marcorelles et al., 2010; Verloes et al., 2014; Yates et al., 2017). In this study with associated callosal agenesis and heterotopia, we have demonstrated an array of underlying pathologies, such as impairments in the proliferation of the progenitor cells, a noticeable reduction of neurons, and growth cones without leading edges in the CGE. We have identified that proteins involved in forming the radial glia and astroglia are impaired. The lack of the neuroepithelial structures identified along the ventricular lining in the *ACTG1* variant substantiates that this can have a knock-on effect on the oligodendrocytes and the microglial formation. The mechanisms of *ACTG1* variants and other variants have recently been described in Ibanez and Andressoo (2016), where researchers have identified variants that were correlated to abnormalities seen in the brain (i.e., basal ganglia and hippocampus) and other organs such as the kidneys (i.e., renal agenesis). In the present study, we demonstrate that there was ACC and neuronal heterotopia that may have been due to malformation of the radial fibers. Additionally, the post-mortem report described a severely hypoplastic kidney.

### The Composition of Neuronal Heterotopia

Previously, Marcorelles et al. (2010) demonstrated that PVWM heterotopia were positive for GABAergic interneurons. The results presented in this study are compatible with (Marcorelles et al., 2010) as we found that in the PVWM the neuronal heterotopia contains calretinin-positive cells. Additionally, we have identified that inside the neuronal heterotopia clusters there are large SNAP-25 positive glutamatergic neurons. Furthermore, we have shown that the neuronal heterotopia are making synaptic vesicles, identified with synaptophysin protein. If the radial glia fails to project from the ventricular zone to the cortical plate, then guidance cues for the glutamatergic neurons would be compromised. This lack of guidance possibly causes these destined cortical neurons to negotiate another destination or to simply “get stuck” causing either obstacles or attraction forces for the migration of the GABAergic neurons. Misplaced neuronal populations can cause lifelong neurological disabilities such as epilepsy and cognitive impairments.

Identifying changes in proteins associated with neuronal heterotopia may improve our understanding of the molecular mechanisms associated with migrational disturbances.

## Ethics Statement

Written and informed parental consent was acquired for post-mortem examination and post-mortem research according to National Health Service United Kingdom and Human Tissue Authority guidelines. Research study ethics was obtained from the National Research Ethics Service (West London), United Kingdom [ethics number, 07/H0707/139; Post-mortem Magnetic Resonance Imaging (MRI) Study of the Developing Brain].

## Author Contributions

RV, VS, CT, HH, and MR conceived and planned the experiments. VS, AM, RV, and AD contributed to the sample preparation. RV carried out the histochemical and immunohistochemical staining and prepared the photomicrographs. AD prepared the representative images from the MRI scans. MH-E requested the whole exome sequencing on the fetus’s DNA-interpreted the results. SL, SY, and MJ contributed to the whole exome sequencing and analysis of the data. RV and MR took the lead in writing the manuscript. All authors provided the critical feedback and helped shape the research, analysis, and the manuscript.

## Conflict of Interest Statement

The authors declare that the research was conducted in the absence of any commercial or financial relationships that could be construed as a potential conflict of interest.

## References

[B1] Achiron R. Achiron A. (2001). Development of the human fetal corpus callosum: a high-resolution, cross-sectional sonographic study. *Ultrasound Obstet. Gynecol.* 18 343–347. 10.1046/j.0960-7692.2001.00512.x 11778993

[B2] Antonucci F. Corradini I. Morini R. Fossati G. Menna E. Pozzi D. (2013). Reduced SNAP-25 alters short-term plasticity at developing glutamatergic synapses. *EMBO Rep.* 14 645–651. 10.1038/embor.2013.75 23732542 PMC3701242

[B3] Bramanti V. Tomassoni D. Avitabile M. Amenta F. Avola R. (2010). Biomarkers of glial cell proliferation and differentiation in culture. *Front. Biosci.* 2 558–570. 10.2741/s85 20036968

[B4] Brazel C. Y. Romanko M. J. Rothstein R. P. Levison S. W. (2003). Roles of the mammalian subventricular zone in brain development. *Prog. Neurobiol.* 69 49–69. 10.1016/s0301-0082(03)00002-9 12637172

[B5] Bruckner G. Brauer K. Hartig W. Wolff J. R. Rickmann M. J. Derouiche A. (1993). Perineuronal nets provide a polyanionic, glia-associated form of microenvironment around certain neurons in many parts of the rat brain. *Glia* 8 183–200. 10.1002/glia.440080306 7693589

[B6] Clowry G. J. (2014). An enhanced role and expanded developmental origins for gamma-aminobutyric acidergic interneurons in the human cerebral cortex. *J. Anat.* 225 2–10. 10.1111/joa.12198 24839870 PMC4580098

[B7] Conde C. Caceres A. (2009). Microtubule assembly, organization and dynamics in axons and dendrites. *Nat. Rev. Neurosci.* 10 319–332. 10.1038/nrn2631 19377501

[B8] Condliffe S. B. Matteoli M. (2011). Inactivation kinetics of voltage-gated calcium channels in glutamatergic neurons are influenced by SNAP-25. *Channels* 5 304–307. 10.4161/chan.5.4.16228 21558797

[B9] De Luca C. Papa M. (2016). Looking inside the matrix: perineuronal nets in plasticity, maladaptiv plasticity and neurological disorders. *Neurochem. Res.* 41 1507–1515. 10.1007/s11064-016-1876-2 26935742

[B10] Edwards T. J. Sherr E. H. Barkovich A. J. Richards L. J. (2014). Clinical, genetic and imaging findings identify new causes for corpus callosum development syndromes. *Brain* 137 1579–1613. 10.1093/brain/awt358 24477430 PMC4032094

[B11] Erzurumlu R. S. Guido W. Molnár Z. (2006). *Development and Plasticity in Sensory Thalamus and Cortex.* New York, NY: Springer.

[B12] Geraldo S. Gordon-Weeks P. R. (2009). Cytoskeletal dynamics in growth-cone steering. *J. Cell Sci.* 122(Pt 20) 3595–3604. 10.1242/jcs.042309 19812305 PMC2758798

[B13] Geraldo S. Khanzada U. K. Parsons M. Chilton J. K. Gordon-Weeks P. R. (2008). Targeting of the F-actin- binding protein drebrin by the microtubule plus-tip protein EB3 is required for neuritogenesis. *Nat. Cell Biol.* 10 1181–1189. 10.1038/ncb1778 18806788

[B14] Gill G. W. Frost J. K. Miller K. A. (1974). A new formula for a half-oxidized hematoxylin solution that neither overstains nor requires differentiation. *Acta Cytol.* 18 300–311. 4135333

[B15] Hetts S. W. Sherr E. H. Chao S. Gobuty S. Barkovich A. J. (2006). Anomalies of the corpus callosum: an MR analysis of the phenotypic spectrum of associated malformations. *AJR* 187 1343–1348. 10.2214/ajr.05.0146 17056927

[B16] Ibanez C. F. Andressoo J. O. (2016). Biology of GDNF and its receptors - relevance for disorders of the central nervous system. *Neurobiol. Dis.* 97(Pt B) 80–89. 10.1016/j.nbd.2016.01.021 26829643

[B17] Ishii K. Kubo K.-I. Endo T. Yoshida K. Benner S. Ito Y. (2015). Neuronal heterotopias affect the activities of distant brain areas and lead to behavioral deficits. *J. Neurosci.* 35 12432–12445. 10.1523/JNEUROSCI.3648-14.2015 26354912 PMC6605399

[B18] Ivanov A. Esclapez M. Ferhat L. (2009a). Role of drebrin A in dendritic spine plasticity and synaptic function: Implications in neurological disorders. *Commun. Integr. Biol.* 2 268–270. 10.4161/cib.2.3.8166 19641748 PMC2717538

[B19] Ivanov A. Esclapez M. Pellegrino C. Shirao T. Ferhat L. (2009b). Drebrin A regulates dendritic spine plasticity and synaptic function in mature cultured hippocampal neurons. *J. Cell Sci.* 122(Pt 4) 524–534. 10.1242/jcs.033464 19174472

[B20] Kadhim H. J. Lammens M. Gosseye S. Gadisseux J. F. Evrard P. (1993). Brain defects in infants with Potter syndrome (oligohydramnios sequence). *Pediatr. Pathol.* 13 519–536. 10.3109/15513819309048240 8372035

[B21] Kato M. (2015). Genotype-phenotype correlation in neuronal migration disorders and cortical dysplasias. *Front. Neurosci.* 9:181 10.3389/fnins.2015.00181 PMC4439546 26052266

[B22] Kolasinski J. Takahashi E. Stevens A. A. Benner T. Fischl B. Zollei L. (2013). Radial and tangential neuronal migration pathways in the human fetal brain: anatomically distinct patterns of diffusion MRI coherence. *NeuroImage* 79 412–422. 10.1016/j.neuroimage.2013.04.125 23672769 PMC4111232

[B23] Kostoviæ I. Sedmak G. Vukšiæ M. Judaš M. (2015). The relevance of human fetal subplate zone for developmental neuropathology of neuronal migration disorders and cortical dysplasia. *CNS Neurosci. Therapeu.* 21 74–82. 10.1111/cns.12333 25312583 PMC6495198

[B24] Lagercrantz H. (2010). *The Newborn Brain : Neuroscience and Clinical Applications* 2nd Edn. New York, NY: Cambridge University Press.

[B25] Leach E. H. (1946). Curtis’ substitute for Van Gieson Stain. *Stain Technol.* 21 107–109. 10.3109/10520294609110359 20993750

[B26] Ledda F. Paratcha G. Sandoval-Guzman T. Ibanez C. F. (2007). GDNF and GFR[alpha]1 promote formation of neuronal synapses by ligand-induced cell adhesion. *Nat. Neurosci.* 10 293–300. 10.1038/nn1855 17310246

[B27] Leviton A. Gressens P. (2007). Neuronal damage accompanies perinatal white-matter damage. *Trends Neurosci.* 3 473–476. 17765331 10.1016/j.tins.2007.05.009

[B28] Marcorelles P. Laquerrière A. Adde-Michel C. Marret S. Saugier-Veber P. Beldjord C. (2010). Evidence for tangential migration disturbances in human lissencephaly resulting from a defect in LIS1, DCX and ARX genes. *Acta Neuropathol.* 120 503–515. 10.1007/s00401-010-0692-z 20461390

[B29] Marin O. Rubenstein J. L. R. (2001). A long, remarkable journey: tangential migration in the telencephalon. *Nat. Rev. Neurosci.* 2 780–790. 10.1038/35097509 11715055

[B30] Mathews K. J. Allen K. M. Boerrigter D. Ball H. Shannon Weickert C. Double K. L. (2017). Evidence for reduced neurogenesis in the aging human hippocampus despite stable stem cell markers. *Aging Cell* 16 1195–1199. 10.1111/acel.12641 28766905 PMC5595679

[B31] Moss J. Gebara E. Bushong E. A. Sánchez-Pascual I. O’Laoi R. El M. (2016). Fine processes of Nestin-GFP–positive radial glia-like stem cells in the adult dentate gyrus ensheathe local synapses and vasculature. *Proc. Natl. Acad. Sci. U.S.A.* 113 E2536–E2545. 10.1073/pnas.1514652113 27091993 PMC4983830

[B32] Paul L. K. (2011). Developmental malformation of the corpus callosum: a review of typical callosal development and examples of developmental disorders with callosal involvement. *J. Neurodev. Disord.* 3 3–27. 10.1007/s11689-010-9059-y 21484594 PMC3163989

[B33] Paul L. K. Brown W. S. Adolphs R. Tyszka J. M. Richards L. J. Mukherjee P. (2007). Agenesis of the corpus callosum: genetic, developmental and functional aspects of connectivity. *Nat. Rev. Neurosci.* 8 287–299. 10.1038/nrn2107 17375041

[B34] Pozzi D. Corradini I. Matteoli M. (2018). The control of neuronal calcium homeostasis by SNAP-25 and its Impact on neurotransmitter release. *Neuroscience* 10.1016/j.neuroscience.2018.11.009 [Epub ahead of print]. 30476527

[B35] Richards S. Aziz N. Bale S. Bick D. Das S. Gastier-Foster J. (2015). Standards and guidelines for the interpretation of sequence variants: a joint consensus recommendation of the American College of Medical Genetics and genomics and the association for molecular pathology. *Genet. Med.* 17 405–424. 25741868 10.1038/gim.2015.30 PMC4544753

[B36] Ross M. E. Swanson K. Dobyns W. B. (2001). Lissencephaly with cerebellar hypoplasia (LCH): a heterogeneous group of cortical malformations. *Neuropediatrics* 32 256–263. 10.1055/s-2001-19120 11748497

[B37] Shu T. Richards L. J. (2001). Cortical axon guidance by the glial wedge during the development of the corpus callosum. *J. Neurosci.* 21 2749–2758. 10.1523/jneurosci.21-08-02749.2001 11306627 PMC6762517

[B38] Sonego M. Oberoi M. Stoddart J. Gajendra S. Hendricusdottir R. Oozeer F. (2015). Drebrin regulates neuroblast migration in the postnatal mammalian brain. *PLoS One* 10:e0126478. 10.1371/journal.pone.0126478 25945928 PMC4422745

[B39] Sturrock R. R. (1978). Development of the indusium griseum. III. an autoradiographic study of cell production. *J. Anat.* 126(Pt 1) 1–6. 649490 PMC1235707

[B40] Sultan K. T. Shi W. Shi S. H. (2014). Clonal origins of neocortical interneurons. *Curr. Opin. Neurobiol.* 26 125–131. 10.1016/j.conb.2014.01.010 24531366 PMC4024342

[B41] Verloes A. Di Donato N. Masliah-Planchon J. Jongmans M. Abdul-Raman O. A. Albrecht B. (2014). Baraitser–Winter cerebrofrontofacial syndrome: delineation of the spectrum in 42 cases. *Eur. J. Hum. Genet.* 23:292. 10.1038/ejhg.2014.95 25052316 PMC4326722

[B42] Volpe J. (2008). *Neurology of the Newborn* 5 Edn. Philadelphia, PA: Saunders Elsevier.

[B43] Vontell R. Supramaniam V. Thornton C. Wyatt-Ashmead J. Mallard C. Gressens P. (2013). Toll- like receptor 3 expression in glia and neurons alters in response to white matter injury in preterm infants. *Dev. Neurosci.* 35 130–139. 10.1159/000346158 23548575 PMC3826123

[B44] Vontell R. Supramaniam V. Wyatt-Ashmead J. Gressens P. Rutherford M. Hagberg H. (2015). Cellular mechanisms of toll-like receptor-3 activation in the thalamus are associated with white matter injury in the developing brain. *J. Neuropathol. Exp. Neurol.* 74 273–285. 10.1097/NEN.0000000000000172 25668563 PMC4327391

[B45] Watrin F. Manent J.-B. Cardoso C. Represa A. (2015). Causes and consequences of gray matter heterotopia. *CNS Neurosci. Therapeu.* 21 112–122. 10.1111/cns.12322 25180909 PMC6495304

[B46] Wonders C. P. Anderson S. A. (2006). The origin and specification of cortical interneurons. *Nat. Rev. Neurosci.* 7:687. 10.1038/nrn1954 16883309

[B47] Yang H. Zhang M. Shi J. Zhou Y. Wan Z. Wang Y. (2017). Brain-specific SNAP-25 deletion leads to elevated extracellular glutamate level and schizophrenia-like behavior in mice. *Neural Plast.* 2017:11. 10.1155/2017/4526417 29318050 PMC5727794

[B48] Yates T. M. Turner C. L. Firth H. V. Berg J. Pilz D. T. (2017). Baraitser-Winter cerebrofrontofacial syndrome. *Clin. Genet.* 92 3–9. 10.1111/cge.12864 27625340

