# Supplementary figures and images for: Post-mortem Characterisation of a Case With an ACTG1 Variant, Agenesis of the Corpus Callosum and Neuronal Heterotopia
Source: Front Physiol. 2019 May 24;10:623. doi: 10.3389/fphys.2019.00623 (PMC6558385; doi:10.3389/fphys.2019.00623)

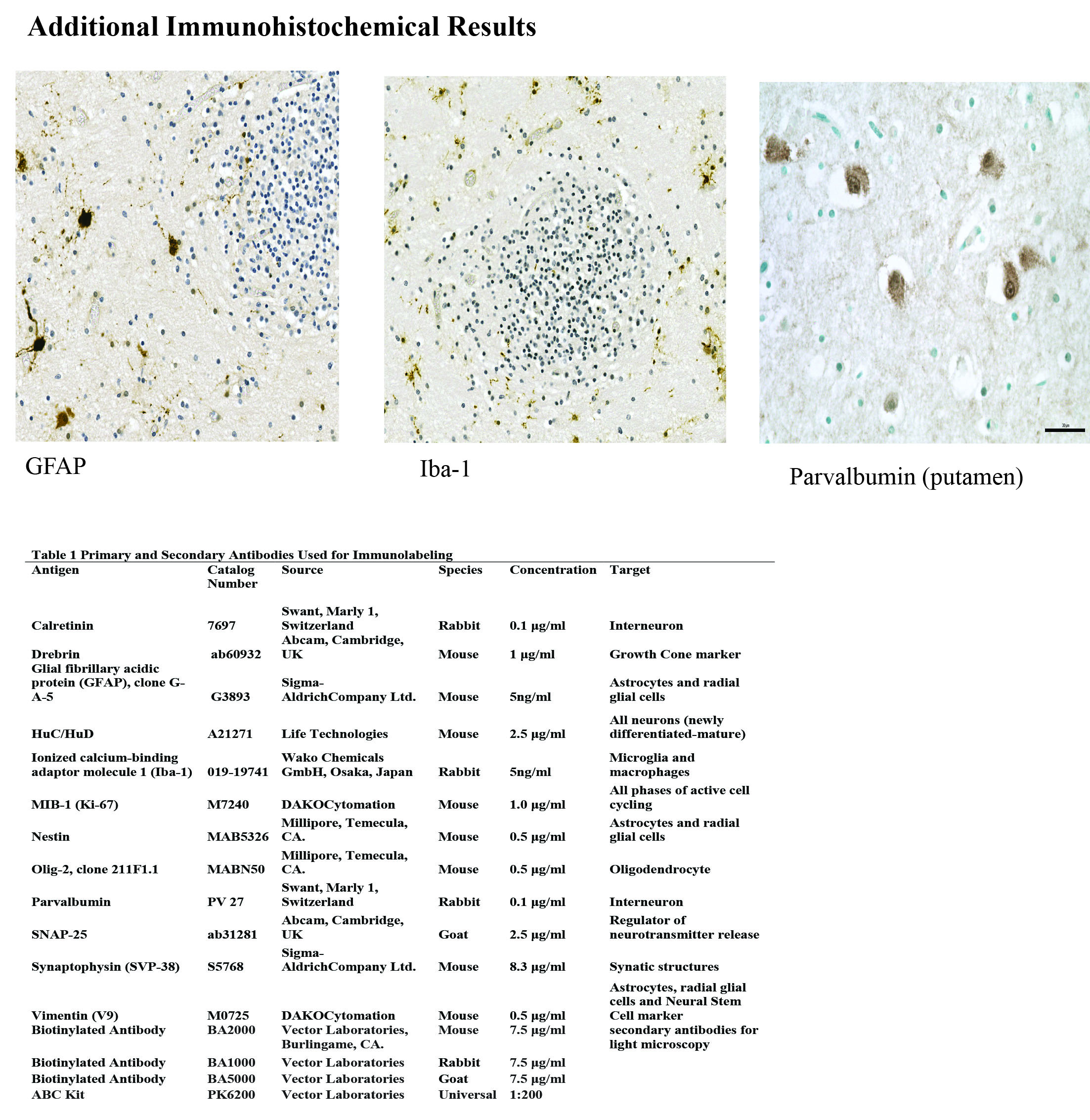

Supplement: Supplementary file 1 [file Image_1.JPEG]
